# Design of epitope-specific probes for sera analysis and antibody isolation

**DOI:** 10.1186/1742-4690-9-S2-P50

**Published:** 2012-09-13

**Authors:** I Georgiev, P Acharya, SD Schmidt, Y Li, D Wycuff, G Ofek, N Doria-Rose, TS Luongo, Y Yang, T Zhou, BR Donald, JR Mascola, PD Kwong

**Affiliations:** 1 Vaccine Research Center/NIAID/NIH, Bethesda, MD, USA; 2 The Scripps Research Institute, La Jolla, CA, USA; 3 National Institutes of Health, Bethesda, MD, USA; 4 Duke University, Durham, NC, USA

## Background

The design of gp120 monomeric probes with modified antigenic profiles that are specific for a target epitope has been successfully used for the isolation of broadly neutralizing HIV-1 antibodies. Existing probes, however, do not possess sufficient specificity and can bind antibodies with undesired properties (e.g., weakly neutralizing antibodies targeting an overlapping epitope). To achieve improved epitope specificity, positive and negative design stages can be incorporated into the probe design process.

## Methods

Here, we apply a combination of structure- and sequence-based methods for improving the epitope specificity of gp120 monomeric probes. Specifically, structure-based redesign using the OSPREY protein design software suite was used to predict gp120 knock-out mutations for selected antibodies. Additionally, using a sequence-based mutual information approach, knock-out mutations were designed by identifying gp120 residues that are predicted to associate with neutralization resistance for a given antibody.

## Results

Using stabilized (Ds12F123) and resurfaced stabilized (RSC3) HXB2 gp120 cores as templates, we designed a set of mutants with improved epitope specificity. In particular, RSC3 (a prototypic probe previously used for the isolation of VRC01 and other CD4-binding-site antibodies) was redesigned to specifically bind CD4-binding-site antibodies that are broadly neutralizing (VRC01, VRC-PG04: positive design) but not moderately/weakly neutralizing (b12, b13, HJ16: negative design). Additionally, CD4i-specific probes were designed by introducing mutations that destabilize binding to the entire class of CD4-binding-site antibodies (negative design), while retaining binding to CD4i antibodies (positive design). The desired epitope specificity of the redesigned probes was confirmed by ELISA binding. The probe design approach was further validated with knock-out mutations for a diverse set of antibodies, including PG9 and 2F5.

## Conclusion

Probes with enhanced epitope specificity can select more precisely for antibodies with desired properties. A set of our redesigned probes are currently being utilized for the isolation of antibodies targeting different epitopes of interest on the HIV-1 Envelope.

